# Predicting Key Events in the Popularity Evolution of Online Information

**DOI:** 10.1371/journal.pone.0168749

**Published:** 2017-01-03

**Authors:** Ying Hu, Changjun Hu, Shushen Fu, Mingzhe Fang, Wenwen Xu

**Affiliations:** Department of Computer and Communication Engineering, University of Science and Technology Beijing, Beijing, China; Beihang University, CHINA

## Abstract

The popularity of online information generally experiences a rising and falling evolution. This paper considers the “burst”, “peak”, and “fade” key events together as a representative summary of popularity evolution. We propose a novel prediction task—predicting when popularity undergoes these key events. It is of great importance to know when these three key events occur, because doing so helps recommendation systems, online marketing, and containment of rumors. However, it is very challenging to solve this new prediction task due to two issues. First, popularity evolution has high variation and can follow various patterns, so how can we identify “burst”, “peak”, and “fade” in different patterns of popularity evolution? Second, these events usually occur in a very short time, so how can we accurately yet promptly predict them? In this paper we address these two issues. To handle the first one, we use a simple moving average to smooth variation, and then a universal method is presented for different patterns to identify the key events in popularity evolution. To deal with the second one, we extract different types of features that may have an impact on the key events, and then a correlation analysis is conducted in the feature selection step to remove irrelevant and redundant features. The remaining features are used to train a machine learning model. The feature selection step improves prediction accuracy, and in order to emphasize prediction promptness, we design a new evaluation metric which considers both accuracy and promptness to evaluate our prediction task. Experimental and comparative results show the superiority of our prediction solution.

## Introduction

**Fig 1:**
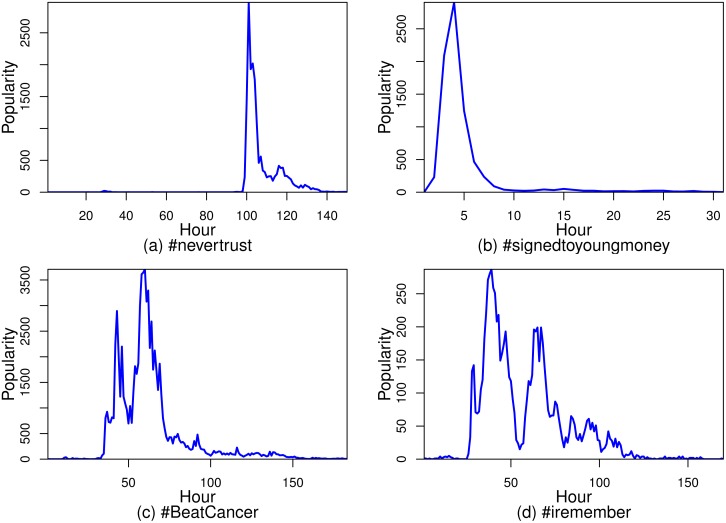
The popularity evolution of Twitter hashtags exhibits rich temporal variation. In (a) and (b), the popularity evolution experiences one huge spike, and the popularity bursts and peaks suddenly. In (c) and (d), the popularity evolution generally follows a rising and falling pattern despite several spikes.

Thanks to the ubiquity of social media sites, massive amounts of online information (e.g. news, videos, pictures, hashtags, etc.) are constantly being produced. Because of many factors, including user interests, real world events, and celebrity involvement, online information exhibits variable popularity evolution [1–3]. Some pieces of online information gain little popularity at first, but burst suddenly, and then fade slowly. Some reach peaks very soon but die an early death. Others experience more variable behaviors [4] (but still approximately follow a rising and falling evolution), as shown in Fig 1. Therefore, the popularity of online information can generally be viewed as a kind of organism that undergoes three key events during its evolution: “burst”, “peak”, and “fade”.

Just like growth, maturation, and aging are defining events in the life of an organism, we think that “burst”, “peak”, and “fade” are representative events in the popularity evolution of online information. If we can predict these events, we can take a full view of the whole popularity evolution and determine the overall popularity trend [5] of online information. In this paper, we propose the prediction of the burst, peak, and fade events in popularity evolution as a new prediction task.

Our work is mainly inspired by the studies of popularity evolution prediction, concerning such fields as volume prediction [6–9] and burst prediction [10–13]. Since most existing work concentrates on predicting future popularity volume (e.g. predicting the value of popularity one day later), this paper considers the problem from another angle: predicting when popularity bursts, peaks, and fades. Recent work has focused on predicting popularity will burst or not [14] or when popularity will burst [13]. To capture the whole evolution, “peak”, and “fade” are equally as important as “burst”. We simultaneously study “burst”, “peak”, and “fade” and view them as a representative summary of popularity evolution.

This paper presents a rigorous study that rises to the two challenges in this task.

**Challenge 1**. The popularity of online information exhibits rich temporal variation and follows various evolution patterns. How can we identify these key events in these circumstances? By using a simple moving average (SMA) we smooth temporal variation to extract the trending line of popularity evolution. Burst, peak, and fade events are then defined from the trend line of popularity evolution.

**Challenge 2**. As we can see in Fig 1, popularity usually bursts and peaks very quickly, which makes predictions more difficult. This requires us to emphasize not only the accuracy but also the promptness of a prediction. On a data set containing approximately 3 million Twitter hashtags and 40 million users, this paper presents a simple solution which consists of the steps of feature extraction, feature selection, and model training by using the selected features. Different types of features, including temporal features, social features, hashtag string features, and topological features, are extracted from hashtags. Features that are highly related with the burst, peak, and fade events are selected through correlation analysis. This step helps to improve prediction accuracy. Experimental results show our solution outperforms other solutions in terms of prediction accuracy. Finally, we construct a new evaluation metric that takes not only accuracy but also promptness into account, and show how to use this new metric to evaluate predictions made at different time points.

We mention the following scenarios where the prediction of burst, peak, and fade events can be used. According to when popularity will burst and fade, online advertisers [15] can decide when to place and remove their advertisements. Caching systems can also decide when to cache and remove webpages. Furthermore, a rumor [16, 17] can be stopped in time if we predict its bursting or peaking time.

The contribution of this paper is three-fold. First, we put forth a new and challenging prediction task—predicting the “burst, peak, and fade” key events. Second, this paper provides a solution and new evaluation metric for this task. Third, this paper finds that the key events are highly correlated with each other after a logarithmic transformation, and that one of the temporal features (the amount of time it takes popularity to reach a certain level once evolution begins) has the most effect on the key events.

The rest of the paper proceeds as follows. Section 2 introduces background and related work. Section 3 presents problem definitions and the characteristics of the three events. A solution is described in Section 4. How to evaluate this prediction task is discussed in Section 5. Section 6 concludes the paper.

## Background and Related Work

Since the emergence of social media sites, a great deal of research interest has arisen in the area of online information popularity [18–20]. Some studies [6, 21, 22] refer to popularity as the attention online information receives from people. Researchers have tried to model and predict how the amount of attention will be devoted over time to a given piece of online information (that is, popularity evolution). Our work relates to the two directions of popularity evolution: popularity evolution patterns and popularity evolution prediction [23, 24].

### Popularity Evolution Pattern

Researchers have found several patterns to characterize how popularity rises and falls during its evolution.

Yang and Leskovec [2] proposed the K-Spectral Centroid (K-SC) clustering algorithm by adopting a time series similarity metric. The algorithm uncovered six popularity evolution patterns according to the rise and fall of popularity. The biggest cluster had a quick rise followed by a monotonic decay and the rate of decay was slightly lower than that of rise. Figueriedo et al. [25] utilized different YouTube data sets: videos on the top lists and videos protected by copyright to analyze how the popularity of individual videos evolved. Copyright protected videos tend to get most of their popularity much earlier during their evolution, often exhibiting a popularity growth characterized by a viral epidemic-like propagation process. In contrast, videos on the top lists tend to experience sudden significant peaks of popularity.

The above work characterizes popularity evolution from a qualitative perspective, while other work has done so from a quantitative perspective. Crane and Sornette [1] used a self-excited Hawkes conditional Poisson process to model the popularity evolution of YouTube videos and showed that the popularity of YouTube videos follows the evolution pattern of a power-law rise and power-law fall. Matsubara et al. [4] stated that real data followed the popularity evolution pattern of an exponential rise and power-law fall and their model (the SpikeM model) which obeyed this pattern fitted real data very well. Yu et al. [26] propose a representation named “phase” to capture the salient rising or falling trend of popularity, which extends the work of Crane and Sornette.

The above studies are the starting point for this paper. Since all these studies demonstrate that the popularity of online information generally experiences a rising and falling evolution, we view the burst, peak, and fade events as a representative summary of popularity evolution.

### Popularity Evolution Prediction

Considerable work has been conducted on predicting future popularity volume. Szabo and Huberman [6] presented a regression prediction model based on the strong linear correlation between the logarithmically transformed popularity of YouTube videos at previous and future times. To predict the bursting and peaking popularity volume, Li et al. [27] developed a popularity prediction solution (SoVP) which recorded viewing-sharing relationships among users to calculate the influence of underlying diffusion structures. He et al. [8] considered two types of sources in the comments of online information: timestamps for obtaining a temporal factor, and usernames for mining potential social influence to model comments as a time-aware bipartite graph to predict future popularity. To predict the final popularity volume of Twitter tweets, Zhao et al. [9] proposed a self-exciting point process model to capture “rich get richer” phenomenon in popularity evolution.

The above work focuses on popularity volume, whereas other studies have focused on whether popularity will burst or not and when popularity will burst. Kong et al. [14] present a binary classification task: will popularity burst in the near feature? They found that the SVM model achieves the best performance in their task. Wang et al. [13] predict when a burst will come. Due to the diverse time spans of popularity evolution, they formulate their problem as a classification problem to predict in which time window burst will appear.

In contrast to most existing work, we consider the three events together and place emphasis on predicting when popularity undergoes these three key events, rather than on future popularity volume.

## Preliminaries

This section first introduces the basic concepts in this paper and discusses how to identify burst, peak, and fade events in popularity evolution. This section then presents our data set and finally discusses the characteristics of the three events.

### Problem Definitions

**Popularity**. By the popularity of a piece of online information we refer to the amount of attention this information receives, such as, the number of views that a video receives, or the number of users discussing a hashtag.

**Popularity Evolution**. Note that most pieces of online information undergo both active and inactive periods [28]. We use the same method as that in [28] to distinguish between both periods: we consider a piece of information inactive if it gains no popularity for 24 hours. To simplify the problem, we shorten popularity evolution to the single active period during which the most popularity volume accumulates. Given the observations of the popularity of a piece of online information *i* over its popularity evolution period *L*_*i*_, *L*_*i*_ ∈ *N*^+^, we define *y*_*i*_(*t*) as the popularity received by the piece of information *i* at time *t*, *t* ∈ {1, 2, 3, …, *L*_*i*_}. The data granularity is set to one hour. For example, *y*_*i*_(10) denotes the popularity received by *i* during the tenth hour. The popularity evolution of *i* is given by the time series {*y*_*i*_(1), *y*_*i*_(2), *y*_*i*_(3), …, *y*_*i*_(*L*_*i*_)}.

**Burst, Peak, and Fade**. Given the popularity evolution of a piece of online information, the qualitative definitions of burst, peak, and fade events are as follows. Directly after popularity undergoes the fastest rate of increase (e.g. the fastest hourly rate of increase) during its evolution, the “burst” event occurs. When popularity reaches the highest value, the “peak” event occurs. Directly after popularity undergoes the fastest rate of decrease, the “fade” event occurs.

**Fig 2:**
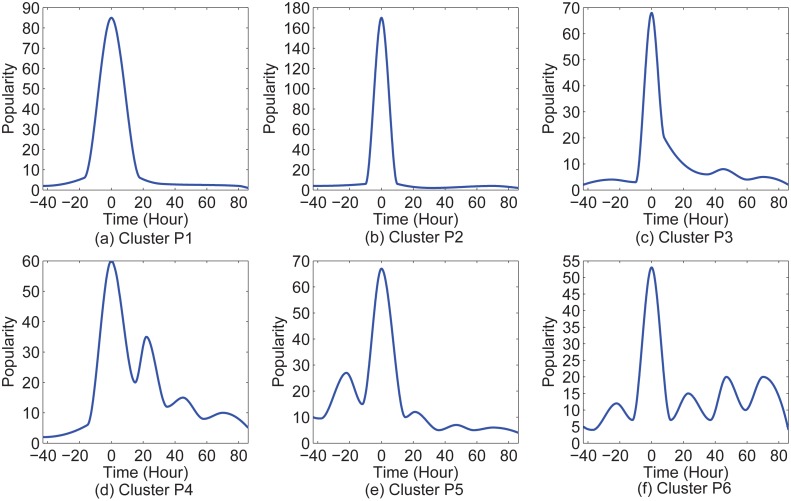
The above six popularity evolution patterns of Twitter hashtags were found by Yang and Leskovec [2]. We consider two categories: Clusters P1, P2, and P3 as one, and Clusters P4, P5, and P6 as the other one. For the first category, there is only one huge spike during popularity evolution. For the second category, there are multiple spikes.

Fig 1 indicates that popularity evolution exhibits rich temporal variation. How can we design a reasonable method that can identify the burst, peak, and fade events in variable evolution and work for all cases of popularity evolution? The six patterns found by Yang and Leskovec [2] can cover almost all cases of popularity evolution, according to Fig 2 [2]. Therefore, we discuss the identification of the events for those six patterns.

In the cases of Clusters P1, P2, and P3, popularity experiences only one huge spike. The burst, peak, and fade events are identified directly from the time series of popularity evolution in the cases of Clusters P1, P2, and P3.

If $T_{i}^{p}\in \left\{1,2,3,\ldots ,L_{i}\right\}$, and $y_{i}\left(T_{i}^{p}\right)$ is the largest element in {*y*_*i*_(1), *y*_*i*_(2), *y*_*i*_(3), …, *y*_*i*_(*L*_*i*_)}, we say the “peak” event of *i* occurs at time point $T_{i}^{p}$.

If $T_{i}^{b}\in \left\{1,2,3,\ldots ,T_{i}^{p}\right\}$, and ($y_{i}\left(T_{i}^{b}\right)-y_{i}\left(T_{i}^{b}-1\right)$) is the largest element in $\left\{y_{i}\left(2\right)-y_{i}\left(1\right),y_{i}\left(3\right)-y_{i}\left(2\right),\ldots ,y_{i}\left(T_{i}^{p}\right)-y_{i}\left(T_{i}^{p}-1\right)\right\}$, we say the “burst” event of *i* occurs at time point $T_{i}^{b}$.

If $T_{i}^{f}\in \left\{T_{i}^{p},T_{i}^{p}+1,T_{i}^{p}+2,\ldots ,L_{i}\right\}$, and ($y_{i}\left(T_{i}^{f}\right)-y_{i}\left(T_{i}^{f}+1\right)$) is the largest element in $\left\{y_{i}\left(T_{i}^{p}+1\right)-y_{i}\left(T_{i}^{p}+2\right),y_{i}\left(T_{i}^{p}+2\right)-y_{i}\left(T_{i}^{p}+3\right),\ldots ,y_{i}\left(L_{i}-1\right)-y_{i}\left(L_{i}\right)\right\}$, we say the “fade” event of *i* occurs at time point $T_{i}^{f}$.

**Fig 3:**
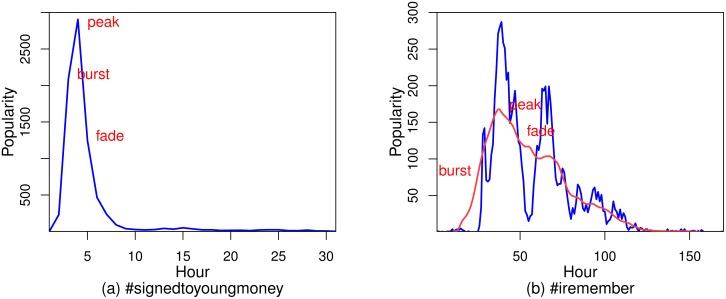
(a) shows the burst, peak, and fade events for Clusters P1, P2, and P3. (b) shows the burst, peak, and fade events for Clusters P4, P5, and P6, and the red line is the smoothed trend which is obtained by using a SMA.

For instance, the popularity evolution of the hashtag #signedtoyoungmoney is represented by the time series {230, 2090, 2905, 1245, 466, 236, 91, 39, 27, 22, 26, …}. So the bursting, peaking, and fading times are the 2nd, 3rd, and 4th hour, respectively, as shown in Fig 3(a).

In the cases of Clusters P4, P5, and P6, popularity experiences one huge spike and several small spikes. These spikes result from people’s daily cycles [6]. Since our key events are more of a representative summary of popularity evolution, they should be identified from the trend of popularity evolution, rather than from the time series with these spikes depending on the time of day. In order to smooth these spikes (in order to mitigate the dependence on the time of day) to obtain the trend of popularity evolution, we use a simple moving average (SMA) with a window length of 24 (because there are 24 hours in one day). After using SMA, we can get a trend line, as shown by the red line in Fig 3(b). The three key events are then identified from a smoothed trend line by using the same method used in the cases of Clusters P1, P2, and P3.

We consider two categories here: Clusters P1, P2, and P3 as one, and Clusters P4, P5, and P6 as the other one. For the sake of simplicity, the two categories can be unified by applying a SMA to the second category and then the same method can be used for both categories in identifications of key events.

### Data Set

**Fig 4:**
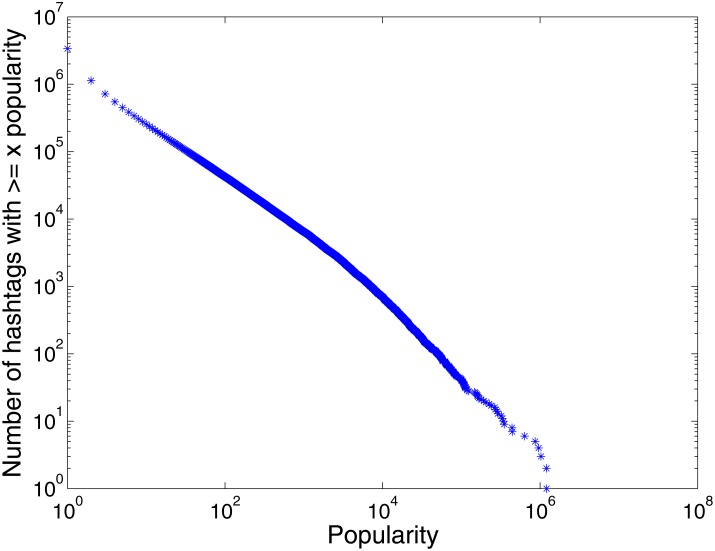
The popularity distribution of all hashtags in our data set.

Our primary data come from a portion of the ‘tweet7’ data set crawled by Yang and Leskovec [2] over a period of 7 months from June to December 2009. (This data set complies with the terms of service for the Twitter website.) The data set comprises 65 million tweets. We identify 3.3 million hashtags in these tweets. Fig 4 shows that the popularity distribution of these 3.3 million hashtags follows a power-law shape. Most of the hashtags in our data set gain very small popularity whereas only a few hashtags gain large popularity. So we select the 3000 most popular hashtags (ranked by the highest value of popularity). The three key events for each of the 3000 hashtags are recorded. All of the following studies are conducted on these selected 3000 hashtags. The statistics of the six popularity evolution patterns are shown in Table 1. (To request our data set, please contact the corresponding author or the owner of the dataset (jure@cs.stanford.edu)).

**Table 1:** Pattern statistics of the 3000 hashtags.

pattern	P1	P2	P3	P4	P5	P6
fraction	15.38%	27.66%	21.92%	11.51%	16.44%	7.09%

### Key Event Characteristics

**Fig 5:**
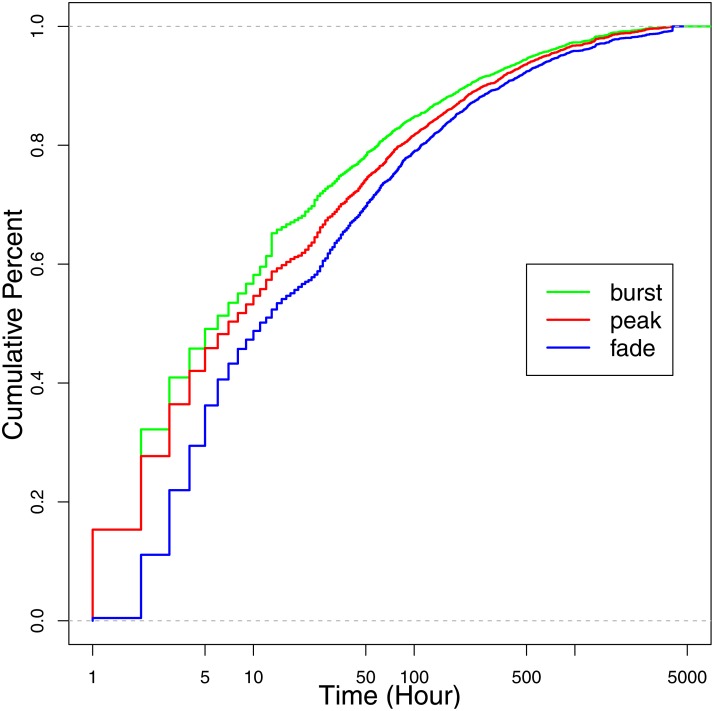
The empirical cumulative distributions of bursting, peaking, and fading times.

After introducing our data set, we take a look at the empirical cumulative distributions of bursting, peaking and fading times (how long it takes these events to occur once popularity evolution begins). Taking the green line in Fig 5 for an example, we logarithmically rescale the horizontal axes in the figures due to the large variances present among the key event times of different hashtags (notice that they range from one to several thousand). For each observed value on the green line, the empirical cumulative distribution shows the fraction of hashtags for which the bursting times are at or below this value. Fig 5 indicates that the bursting times of about 60% of hashtags are within the first ten hours during popularity evolution. Some burst events even occur in the first hour. Bursting in such a short time makes predictions more difficult and challenging. Furthermore, the fading times of over 50% of hashtags are also within the first ten hours. Therefore over 50% of popular hashtags undergo a short popularity evolution: their popularity bursts suddenly, peaks very soon, and then fades quickly.

**Fig 6:**
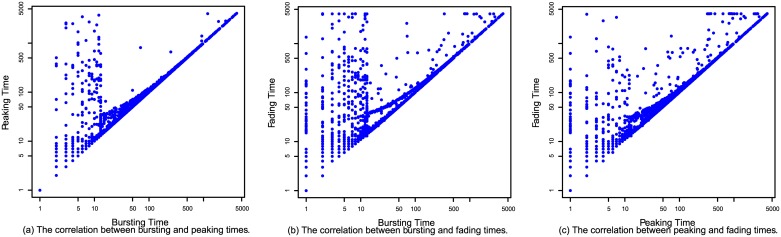
(a) shows the relationship between the burst and peak events. (b) shows the relationship between the burst and fade events. (c) shows the relationship between the peak and fade events.

Since the three key events are studied simultaneously in this paper, we are interested in whether there is any relationship among them. For this, we plot peaking time versus bursting time (Fig 6(a)), fading time versus bursting time (Fig 6(b)), and fading time versus peaking time (Fig 6(c)) for each hashtag. The horizontal and vertical axes are logarithmically rescaled. It can be observed in Fig 6(a) and 6(b) that the data form two major clusters: one loose cluster in the left side of the graph, and a tighter cluster along the main diagonal. The data points along the main diagonals undergo peak and fade events quickly after bursting. The data points in the left side of Fig 6(a) and 6(b) undergo burst events in a short time (in the first ten hours), but it takes them a long time to undergo peak and fade events after bursting. The separation of the two clusters is not clear in Fig 6(c), because peaking times are not as early as bursting times. Most data points in Fig 6(c) are along the main diagonal.

Furthermore, to quantify the strength of the correlations in Fig 6, we measure the Pearson correlation coefficients among the three events. Table 2 shows the Pearson correlation coefficients (PCC) between the logarithmically transformed event times, and for comparison also the correlations between the untransformed event times. Logarithmically transformed event times show stronger correlations. Because of the strong correlations among the three events, this paper utilizes the same method to simultaneously predict burst, peak, and fade events for each hashtag.

**Table 2:** The Pearson correlation coefficients among the key event times.

	burst-peak	burst-fade	peak-fade
log	0.94	0.87	0.94
non-log	0.90	0.71	0.84

## Solution

As we mentioned in Section 3.3, the key events usually occur in a short time, which requires us to make predictions promptly. Predicting the key events simultaneously and not separately facilitates a prompt prediction. For example, we do not have to predict a fade event until the peak event occurs. It is reasonable to predict all three events by the same method at the same time for each piece of online information. If a prediction performs well for a burst event, it should also perform well for the peak and fade event because of the strong correlations among the three events. In this section we first discuss a suitable time to make predictions. The prediction method of this paper relies on the following steps: feature extraction, feature selection, and use of the SVR (Support Vector Regression) model fed by selected features.

### Determining Prediction Times

According to Fig 5, bursting times vary significantly, ranging from several hours to several weeks. It is not reasonable to wait the same time to make predictions for different kinds of popularity evolution. For those requiring several hours to burst, waiting several days results in a useless prediction. For those requiring several weeks to burst, waiting several hours cannot give us sufficient information to make a prediction. So how can we decide when to make the prediction for each individual hashtag without knowing if it will take hours or weeks to burst?

**Fig 7:**
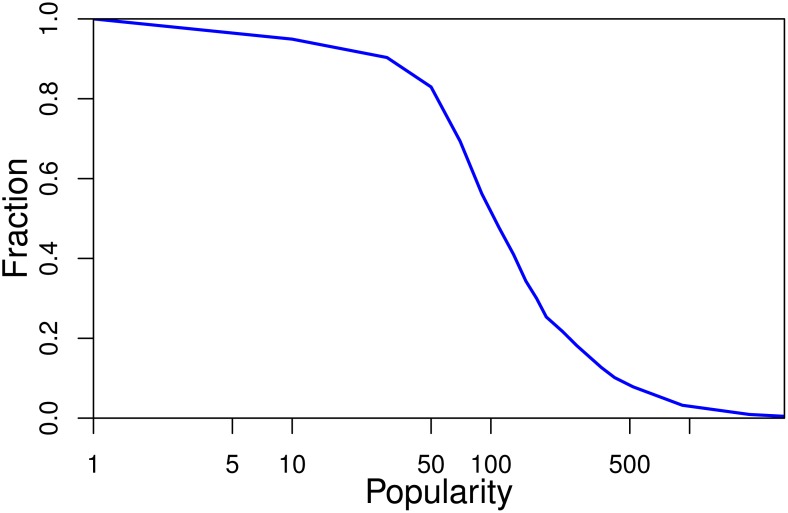
The fraction of hashtags for which popularity reaches the corresponding x value before the “burst” event.

As indicated in Fig 1(a), for those hashtags requiring a long time to burst, their popularity usually remains at a very low value at the beginning, like {1, 1, 2, 3, 0, 0, 2, …}. Once their popularity reaches a certain level, their popularity will increase at a faster rate. This behavior can be explained by the “rich get richer” phenomenon [29]. We therefore propose the idea that predictions are triggered once popularity reaches a certain level, e.g. once popularity reaches 20. Fig 7 shows the fraction of hashtags for which popularity reaches the corresponding x value before the “burst” event. The x axis in Fig 7 is logarithmically transformed. It is easy to understand that this fraction decreases with an increasing value of popularity, as indicated by Fig 7. Hence, predictions for most hashtags should be made before popularity reaches *σ*. According to Fig 7, for about 90% of hashtags, popularity reaches 40 before the “burst” event. For about 85% of hashtags, popularity reaches 50 before the “burst” event. Therefore, *σ* is set to 40 for our data set.

### Features for Predictions of the Key Events

**Temporal Features**. Previous work [29] has suggested that popularity evolves according to the “rich-get-richer” phenomenon, which means that a hashtag receives new popularity at a rate proportional to the value of popularity it has already received. Hence, we deduce that the current cumulative popularity (cumuPopularity) may have impact on the bursting, peaking, and fading times. Another temporal feature is the time of day when the popularity evolution of a hashtag begins (beginningTime). This feature is taken into account because we believe that this feature affects the value popularity will reach in the next hour, which itself affects the event times. For example, a hashtag that starts its popularity evolution at 9 p.m. will receive more popularity in the next hour than a hashtag that starts its popularity evolution at 3 a.m. In the former case, popularity may burst quickly. But it may take a longer time for popularity in the latter case to burst. The last temporal feature is the amount of time it takes popularity to reach a certain level (e.g. 20) once evolution begins (levelTime), which is also the amount of time it should take for predictions to be triggered.

**Social Features**. We assume that celebrity involvement in discussing a hashtag accelerates the occurrences of the key events. We calculate the number of celebrities (celebrityCount) and the overall sum and maximum of the numbers of their followers (fanTotal, fanMax).

**Hashtag String Features**. We manually separate hashtag strings into individual words and count the number of individual words (wordCount). For example, #alovelikethisisonyourside is interpreted as “a love like this is on your side”, so this hashtag has eight individual words. Hashtags that have no clear meaning, like #abcdefg and #bbmg, are considered as one word. Another feature (stringLength) we extract from a hashtag string is the number of letters in the hashtag.

**Topological Features**. We also pay attention to the features of the topology networks formed by the users discussing each hashtag [30]. We denote by *N*_*i*_(*t*) = (*V*_*i*_(*t*), *E*_*i*_(*t*)), the cumulative evolving network for hashtag *i* at hour *t*, *t* ∈ {1, 2, 3, …, *L*_*i*_}. The vertex set *V*_*i*_(*t*) of *N*_*i*_(*t*) is the set of all users who have tweeted on hashtag *i* in hours 0 through *t*. An edge between vertex *u* and vertex *v* is added to *E*_*i*_(*t*) if *u* and *v* have a follower-following relationship, *u* ∈ *V*_*i*_(*t*) and *v* ∈ *V*_*i*_(*t*). The follower-following relationships come from a data set collected by Kwak et al. [28] during the same time period that the ‘tweet7’ data set was collected. Several topological features are extracted from *N*_*i*_(*t*), including average node degree (degreeAverage), maximum node degree (degreeMax), global clustering coefficient (ccGlobal), average local clustering coefficient (ccAverage), and the number of nodes in the largest connected component (lccNodeCount).

### Feature Selection

**Fig 8:**
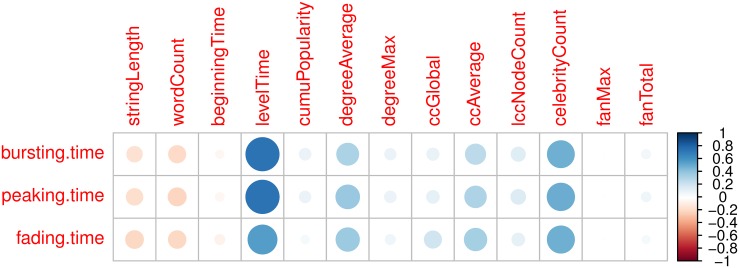
The pairwise correlation between the corresponding event time and feature.

Before feeding the SVR model the above features (extracted once popularity reaches a certain level, e.g. 20), we perform feature selection by removing irrelevant and redundant features. Doing so can unburden the training process and help us understand which feature best captures the bursting, peaking, and fading time. First, we investigate the Pearson correlation between each feature and each event time to remove irrelevant features. Each cell of Fig 8 shows the pairwise correlation between the corresponding event time and feature. The correlation’s magnitude is represented by the circle symbol, and its sign is represented by colors, with red used for negative values and blue for positive values. Fig 8 indicates that levelTime and celebrityCount have high correlations with bursting, peaking, and fading time. beginningTime and fanMax are not related with the event times. Note that each row in Fig 8 looks very similar, which also indicates the three event times are highly correlated with each other. After removing irrelevant features, the remaining ones are levelTime, celebrityCount, degreeAvarage, and ccAverage.

**Fig 9:**
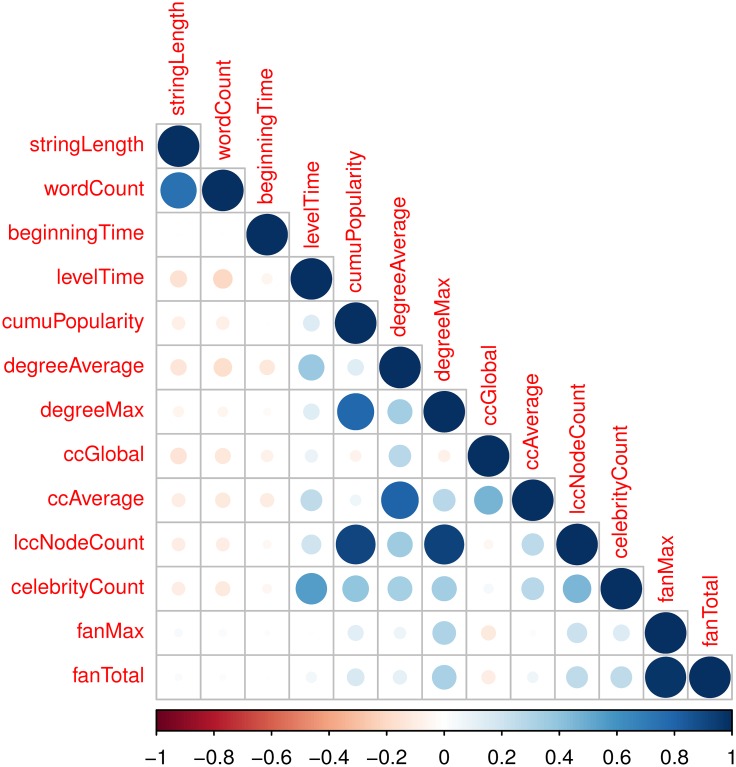
The pairwise correlations among features.

Second, we study the Pearson correlations among these features to remove redundant features. Fig 9 shows the correlation matrix for all features. Some of the features have high pairwise correlations, such as lccNodeCount and degreeMax, lccNodeCount and cumuPopularity, fanMax and fanTotal. After removing irrelevant and redundant features according to Figs 8 and 9, the remaining ones are levelTime, and ccAverage.

## Evaluation

This section discusses how this task is evaluated. First, traditional evaluation metrics, which only consider accuracy, are adopted to evaluate our solution. We compare our solution with others in terms of traditional evaluation metrics. Second, a new evaluation metric, which considers both accuracy and promptness, is specifically designed for this task.

### Experimental setup

According to the tips on practical use of the SVR model [31], we first try small and large values for the general penalizing parameter (*C*) with exponentially growing sequences, like 10^0^, 10^1^, 10^2^, 10^3^, 10^4^, then decide which are better for the data by cross validation minimizing MAPE, and finally try several kernel parameter *γ*s (like 10^0^, 10^−1^, 10^−2^, 10^−3^, 10^−4^) for the better *C*s. For our data set, *C* is set to 1000, and *γ* is set to 0.0001. In the following two-sided paired t-tests we conduct to compare our solution with other solutions, the sample size (*n*) is 3000.

### Traditional Evaluation

#### Overall Performance

The overall performance of our solution is evaluated in terms of the minimum, quartiles, and maximum values of prediction errors (Eq 1) and relative errors (Eq 2). We perform a 5-fold cross-validation. We evaluate predictions made at different time points (e.g. the time points once popularity reaches 10, 20, 30, and 40).

$$\begin{array}{c} Error_{i}=\left|t_{i}^{\prime }-t_{i}\right| \end{array}$$ (1)

$$\begin{array}{c} RelativeError_{i}=\frac{|t_{i}^{\prime }-t_{i}|}{t_{i}} \end{array}$$ (2)

where $t_{i}^{\prime }$ is the predicted event time of the hashtag *i*, and *t*_*i*_ is the actual event time.

Note that in some cases relative errors are larger than 1 because of low actual value and high predicted value. Table 3 gives the fractions of relative errors exceeding 1 for different predictions. In the cases where burst and peak predictions are triggered when popularity reaches 10, the fraction values are about 30%. These high fraction values result from bad prediction performance and low actual values. In accordance with standard procedure in the prediction field, we set relative errors to 1 when they exceed 1.

**Table 3:** Fractions of relative errors exceeding one for the predictions triggered once popularity reaches 10, 20, 30, and 40.

	10	20	30	40
burst	31.56%	7.44%	2.76%	2.99%
peak	30.10%	19.81%	7.52%	1.15%
fade	5.37%	1.15%	0.92%	1.13%

Table 4 shows the minimum, quartile (Q1 (the first quartile), Q2 (median), and Q3 (the third quartile)), and maximum error values for predictions of the three event. We can make the following observations. (1) Overall errors decrease with prediction time for predictions of all three events. This is consistent with the intuition that the later we predict, the more accurate the prediction is. (2) Taking the predictions triggered once popularity reaches 20 for an example. For 50% of hashtags, the difference between the predicted bursting (peaking) time and the actual bursting (peaking) time is less than one hour. For 75% of hashtags, this time difference is less than two hours. For 50% of hashtags, the difference between the predicted fading time and the actual fading time is less than one hour. For 75% of hashtags, this time difference is less than for hours. (3) The errors for fade event predictions are usually not as low as those for burst and peak event predictions, which can be explained by Fig 8: the correlations between the selected features and fading times are not as high as those between the selected features and bursting and peaking times. (4) The maximum error values for all three event predictions are large (greater than 200 hours), which results from the points not along the main diagonals in Fig 6.

**Table 4:** The minimum, quartile, and maximum error values for predictions of the three events.

key event	burst					peak					fade				
stats	min	Q1	Q2	Q3	max	min	Q1	Q2	Q3	max	min	Q1	Q2	Q3	max
10	0	1	1	2	174	0	1	1	3	175	0	1	2	7	257
20	0	0	1	2	171	0	1	1	2	169	0	1	1	6	247
30	0	0	0	2	175	0	0	1	2	175	0	1	1	5	249
40	0	0	0	2	178	0	0	1	2	178	0	1	1	4	258

**Fig 10:**
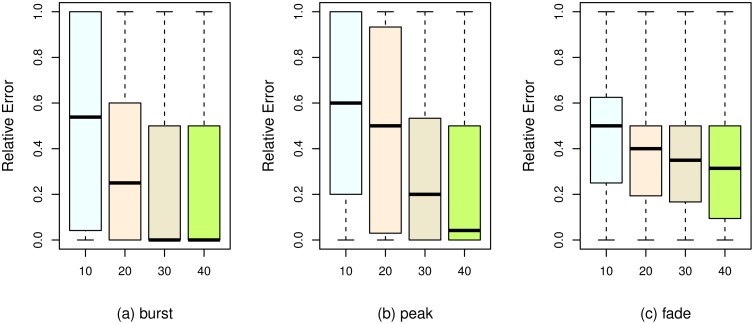
The minimum, quartile, and maximum relative error values for predictions of the three events.

Relative errors are presented in Fig 10. The box-and-whisker plot shows the minimum, quartiles, and maximum error values. The bottom and top of the box are the first and third quartile error values, respectively, and the band inside the box is the median error value. The upper and lower whiskers are the maximum and minimum error values, respectively. The position of the median band becomes lower as prediction time increases, which indicates that the overall prediction performance improves as prediction time increases. The third and fourth boxes in Fig 10(a) show that the median value of relative errors is zero if burst event predictions are triggered once popularity reaches 30 or 40. The third and fourth boxes in Fig 10(b) show that the median values of relative errors are about 0.2 and 0.05 if peak event predictions are triggered once popularity reaches 30 and 40, respectively. The third and fourth boxes in Fig 10(c) show that the median values of relative errors are about 0.3 and 0.35 if fade event predictions are triggered once popularity reaches 30 and 40, respectively.

#### Comparison

To validate the effectiveness of our solution, this paper compares our solution with the following solutions in terms of (1-RelativeError). We also perform 5-fold cross-validations.

**Using all features (AF)**. To show the efficiency of performing the feature selection, we compare our solution using only the selected features with another solution using all the features.

**Using Bayesian linear regression (BLR)**. We replace the SVR model in our solution with another machine learning model known as Bayesian linear regression.

**SpikeM**. Due to the fact that predicting the burst, peak, and fade events is a new prediction task, most of the existing models for popularity evolution prediction are not capable of solving this task. To compare our solution with existing work, we choose a model which can solve this task but is not specialized for it, the SpikeM model [4]. We train the SpikeM model by using popularity data up to the time of prediction to obtain the whole predicted popularity evolution. Then the three event times can be inferred from the whole predicted popularity evolution.

**Fig 11:**
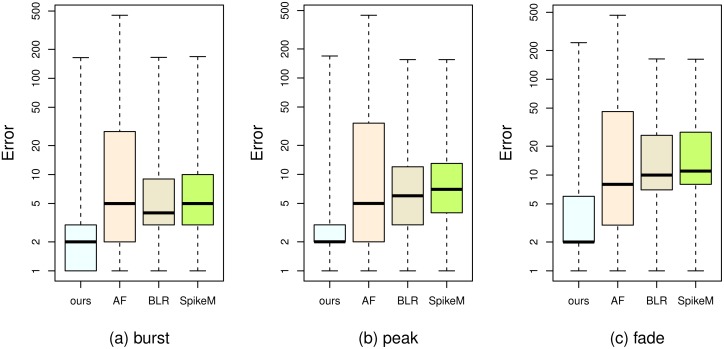
The minimum, quartile, and maximum relative error values for predictions of the three events.

The first and second boxes in each subfigure of Fig 11 and Table 5 show the comparison between our solution and the solution using all features. For better visualization in Fig 11, each prediction error is increased by one hour, and then presented on the logarithmically rescaled vertical axis. For predictions of all three events, we can see that the median error values resulting from using the selected features are about two or three hours lower than those resulting from using all the features. We conduct a significance test (Table 5) to further compare, in terms of (1−*RelativeError*), our solution with the solution using all the features. $\bar{d}$ denotes the mean of (1−*RelativeError*) differences. *V* denotes the variance of (1−*RelativeError*) differences. *p* denotes the p-value. *ES* denotes the effect size. *CI* denotes the confidence interval. According to a two-sided paired t-test for the difference in means $\bar{d}=0.26$ (with the unbiased estimate of the population variance *V* = 0.32), burst event predictions using only the selected features statistically significantly outperform burst event predictions using all the features (*p* < 0.05, *ES* = 0.4634, 95% *CI*[0.2072, 0.3132]). This is similarly true for peak and fade event predictions. The reason for the better performance of our solution is that the irrelevant and redundant features disturb the learning process of the SVR model, and removing the irrelevant and redundant features helps improve the prediction results.

**Table 5:** The significance test of comparing our solution with the solution using all features.

	$\bar{d}$	*V*	*ES*	*p*	*CI*
burst	0.26	0.32	0.46	0.00	[0.21, 0.31]
peak	0.15	0.26	0.30	0.00	[0.10, 0.20]
fade	0.27	0.15	0.71	0.00	[0.24, 0.31]

The first and third boxes in each subfigure of Fig 11 and Table 6 show the comparison between our solution and the solution using the BLR model. For burst and peak predictions, the median error values resulting from using our solution are about two or three hours lower than those resulting from using the BLR model. For fade predictions, the median error value resulting from using our solution is about seven hours lower than that resulting from using the BLR model. We conduct a significance test (Table 6) to further compare our solution with the solution using the BLR model. According to a two-sided paired t-test for the difference in means $\bar{d}=0.35$ (with the unbiased estimate of the population variance *V* = 0.26), our solution statistically significantly outperforms the solution using the BLR model (*p* < 0.05, *ES* = 0.69, 95% *CI*[0.31, 0.40]). This is similarly true for peak and fade event predictions.

**Table 6:** The significance test of comparing our solution with the solution using the BLR model.

	$\bar{d}$	*V*	*ES*	*p*	*CI*
burst	0.35	0.26	0.69	0.00	[0.31, 0.40]
peak	0.27	0.22	0.57	0.00	[0.23, 0.31]
fade	0.43	0.17	1.05	0.00	[0.39, 0.47]

The first and fourth boxes in each subfigure of Fig 11 and Table 7 show the comparison between our solution and the SpikeM model. For burst and peak predictions, the median error values resulting from using our solution are about three hours lower than those resulting from using the SpikeM model. For fade predictions, the median error value resulting from using our solution is about eight hours lower than that resulting from using the SpikeM model. We conduct a significance test (Table 7) to further compare our solution with the SpikeM model. According to a two-sided paired t-test for the difference in means $\bar{d}=0.44$ (with the unbiased estimate of the population variance *V* = 0.25), our solution statistically significantly outperforms the SpikeM model (*p* < 0.05, *ES* = 0.87, 95% *CI*[0.39, 0.49]). This is similarly true for peak and fade event predictions. The main reason for the non-optimal performance of the SpikeM model is that the SpikeM model only considers popularity data, and popularity data are sparse and do not contain enough information to learn the parameters of the SpikeM model due to the quick occurrence of the key events.

**Table 7:** The significance test of comparing our solution with the solution using the SpikeM model.

	$\bar{d}$	*V*	*ES*	*p*	*CI*
burst	0.44	0.25	0.87	0.00	[0.39, 0.49]
peak	0.31	0.22	0.65	0.00	[0.26, 0.35]
fade	0.44	0.17	1.07	0.00	[0.41, 0.48]

### New Evaluation

As we discussed in Section 3.3, some of key events occur in a very short time once popularity evolution begins. Because of this special characteristic, we argue that traditional evaluation metrics (e.g. error, relative error, RMSE, and MAPE, etc.) have a flaw in evaluating this task and that the promptness of a prediction should be taken into account. For instance, we have two hashtags, say A and B. The predictions for the two hashtags are triggered at the 3rd and 5th hours. The prediction results for A and B are both (5, 6, 7). That is, we estimate that A’s and B’s popularity will burst at the 5th hour, peak at the 6th hour, and fade at the 7th hour. The actual results for A and B are both (4, 5, 6). The prediction accuracies are the same for A and B, but the prediction for B is triggered after the popularity bursts. From an application point of view, it is meaningless to make a key event prediction after the key event already occurs. Still taking A and B for an example, this time we make predictions at 2nd and 3rd hours. Their predicted and actual results are the same as those in the first example. This time the predictions for both hashtags are triggered before the key events. From the perspective of stopping rumors and controlling information diffusion, we have two hours left to take actions for A but only one hour for B. Hence, the traditional metrics that only take accuracy into account are not enough. We need a new evaluation metric which considers not only accuracy but also promptness.

This new evaluation metric is constructed like an F1 score. It has two ingredients: correctness score (CS, given in Eq 3) and promptness score (PS, given in Eq 4), so we call this new evaluation metric balanced CP score. A balanced CP score balances accuracy and promptness. Given the results of a key event prediction, its balanced CP score is given in Eq 5 according to the following rules.

**Rule 1**. In some cases correctness scores (1-RelativeError) are lower than 0 because relative errors are higher than 1, as we discussed in Section 5.1.1. We set correctness scores in these cases to 0.

**Rule 2**. If a prediction is triggered before popularity evolution, the corresponding promptness score is set to 1. If a prediction is triggered after a key event time, the corresponding promptness score is set to 0.

$$\begin{array}{c} CS_{i}=\left\{\begin{array}{c} 1-\frac{|t_{i}^{\prime }-t_{i}|}{t_{i}},\;\left|t_{i}^{\prime }-t_{i}\right|\leq t_{i}\\ 0,\;|t_{i}^{\prime }-t_{i}|>t_{i} \end{array}\right. \end{array}$$ (3)

$$\begin{array}{c} PS_{i}=\left\{\begin{array}{c} \frac{t_{i}-t_{i}^{\prime \prime }}{t_{i}},\;t_{i}^{\prime \prime }\leq t_{i}\\ 0,\;t_{i}^{\prime \prime }>t_{i} \end{array}\right. \end{array}$$ (4)

$$\begin{array}{c} BalancedCPScore_{i}=\frac{2CS_{i}*PS_{i}}{CS_{i}+PS_{i}} \end{array}$$ (5)

where $t_{i}^{\prime \prime }$ is the key event prediction time of hashtag *i*.

**Fig 12:**
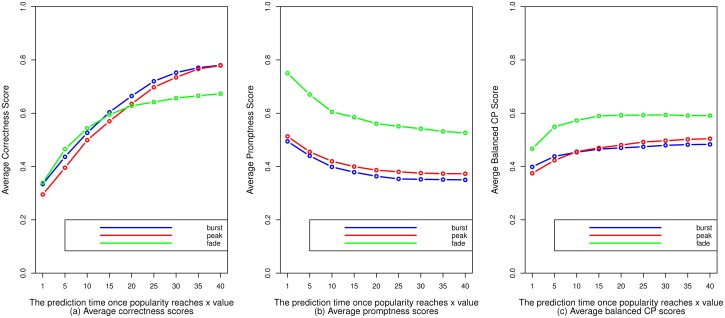
The correctness scores, promptness scores, and balanced CP scores for predictions of the three events.

We use the average of balanced CP scores to evaluate the predictions at different time points. We conduct a 5-fold cross-validation. According to Fig 12(a), for predictions of all three events, the average correctness score increases with prediction time. The reason for this is that the later we make predictions, the stronger correlations are between the selected features and the event times. In Table 4, errors for fade event predictions are larger than errors for peak event predictions, but in Fig 12, the average correctness scores for fade event predictions made at earlier time points are higher than those for peak event predictions. This is because actual fading times are larger than actual peaking times and correctness scores sometimes become higher when actual values are larger. According to Fig 12(b), for predictions of all three events, the average promptness score decreases with prediction time. It is obvious that the later we predict, the lower the values of promptness scores are. Burst event predictions are not as prompt as the peak event predictions, because a burst event always occurs after the corresponding peak event. According to Fig 12(c), balanced CP score tends to become saturated as prediction time increases for predictions of all three events.

## Conclusions

In this paper we bring forward a new and challenging prediction task in the field of popularity evolution: predicting when the “burst”, “peak”, and “fade” key events occur. The challenges of identifying the events in different popularity evolution patterns with high variation, and making accurate yet prompt predictions, are addressed for this task. This paper presents a solution based on the characteristics of the events. Comparative results show that our solution outperforms three other solutions in terms of accuracy. We design a new evaluation metric (balanced CP score) and show how to use this metric to evaluate the quality (both accuracy and promptness) of predictions at different times. Furthermore, we find that the popularity of more than half the hashtags in our selected data set bursts suddenly, peaks very soon, and then fades quickly, that these events have strong correlations with each other, and that the levelTime feature has the most effect on this prediction task compared to other features.

Predictions concerning those points not along the main diagonals in Fig 6 need to be improved in future work. There is another interesting area that we did not explore here: What characteristics do the two clusters in Fig 6 have? We have a rough look into these two clusters. We find that the hashtags in the left cluster tend to have more specific meanings with longer strings, like #lieswomentell, #WorldsThinnestBooks, and #itsnotgonnawork, while the other hashtags are vaguer, like #mnf, #Packers, and #eBay. Our future research will address this question as well.
